# Safety of Transseptal Puncture for Access to the Left Atrium in Infants and Children

**DOI:** 10.1007/s00246-020-02530-9

**Published:** 2021-01-16

**Authors:** Matthias J. Müller, David Backhoff, Heike E. Schneider, Jana K. Dieks, Julia Rieger, Ulrich Krause, Thomas Paul

**Affiliations:** Department of Pediatric Cardiology and Intensive Care Medicine, Georg August University Medical Center, Robert-Koch-Str. 40, 37075 Göttingen, Germany

**Keywords:** Transseptal puncture, Infants, Children, Congenital heart defect

## Abstract

Transseptal puncture (TSP) is a standard procedure to obtain access to the left heart. However, data on TSP in infants and children particularly with congenital heart defects (CHD) is sparse. Safety and efficacy of TSP in infants and children < 18 years with normal cardiac anatomy and with CHD were assessed. 327 TSP were performed in a total of 300 individuals < 18 years from 10/2002 to 09/2018 in our tertiary pediatric referral center. Median age at TSP was 11.9 years (IQR 7.8–15; range: first day of life to 17.9 years). 13 subjects were < 1 year. Median body weight was 43.8 kg (IQR 26.9–60; range: 1.8–121 kg). CHD was present in 28/327 (8.6%) procedures. TSP could be successfully performed in 323/327 (98.8%) procedures and was abandoned in 4 procedures due to imminent or incurred complications. Major complications occurred in 4 patients. 3 of these 4 subjects were ≤ 1 year of age and required TSP for enlargement of a restrictive atrial septal defect in complex CHD. Two of these babies deceased within 48 h after TSP attempt. The third baby needed urgent surgery in the cath lab. Pericardial effusion requiring drainage was noted in the forth patient (> 1 year) who was discharged well later. Minor complications emerged in 5 patients. The youngest of these individuals (0.3 years, 5.8 kg) developed small pericardial effusion after anterograde ballon valvuloplasty for critical aortic stenosis. The remaining 4/5 patients developed small pericardial effusion after ablation of a left-sided accessory atrioventricular pathway (6.1–12.2 years, 15.6–34.0 kg). TSP for access to the left heart was safe and effective in children and adolescents > 1 year of age. However, TSP was a high-risk procedure in small infants with a restrictive interatrial septum with need for enlargement of interatrial communication.

## Introduction

Since introduction into clinical practice in 1959, transseptal puncture (TSP) under fluoroscopic guidance using the Brockenbrough approach is a common procedure to obtain access to the left heart during cardiac catheterization. TSP offers the opportunity to a wide spectrum of diagnostic and interventional left heart procedures like catheter ablation, percutaneous mitral valvuloplasty or clip repair and left atrial appendage occlusion [1, 2]. The target for TSP is the fossa ovalis which is typically localized in the center of the interatrial septum. Due to anatomical variations of the fossa ovalis, TSP may sometimes be challenging. Care must be taken to avoid potentially life-threatening complications as pericardial effusion and tamponade [2]. It is of note, that elective TSP in infants and children with normal cardiac anatomy has been described as feasible and safe with a low complication rate (0.3–2.3%) [3–5] comparable with the complication rate of TSP in adults [6]. It is not yet clear if these results can be adopted for children with urgent need of TSP especially in small infants with congenital heart defects. The purpose of the present study was to examine the risk of TSP in a remarkable number of infants and children < 18 years with normal heart anatomy and congenital heart defects (CHD).

## Patients and Methods

For the present study, data of all infants and children with transseptal access to the left atrium (LA) via transseptal puncture (TSP) during cardiac catheterization in our tertiary pediatric referral center between 10/2002 and 09/2018 were collected. Subjects with a patent formen ovale or an atrial septal defect allowing catheter access to the LA were not included into this study. Informed consent had been obtaiend from the legal guardians of all individuals. The study had been approved by the local scientific committee of the Childen´s Hospital of Georg-August-University Medical Center, Göttingen, Germany.

A total of 327 TSP were performed in 300 subjects < 18 years of age. Median age was 11.9 years (IQR 7.8–15 years; range: first day of life to 17.9 years). 12 subjects were ≤ 1 year of age. Median body weight was 43.8 kg (IQR 26.9–60 kg; range: 1.8–121 kg) and median height was 154 cm (IQR 129–168 cm; range: 40–190 cm). Congenital heart defects were present in 28/327 (8.6%) procedures (Table 1).

**Table 1:** Baseline characteristic of patients (*n* = 327)

		All (*n* = 327)	TSP for ablation (*n* = 305)	TSP for hemodynamic intervention (*n* = 8)	TSP for diagnostic purpose (*n* = 14)
Median age	Years (IQR)	11.9 (7.8–15)	12.1 (8.5–15.1)	0.3 (0.1–0.7)	5.8 (3.2–14.7)
Median body weight	kg (IQR)	43.8 (26.9–60.5)	44.7 (29.6–60.5)	4.6 (3.0–7.3)	17.0 (12.8–53.3)
Median height	cm (IQR)	154 (129–168)	155 (129–168)	58 (51–69)	114 (94–166)
Congenital heart defects					
None	*N* (%)	299 (91.4%)	298 (98%)	0 (0%)	1 (7.1%)
VSD	*N* (%)	1 (0.3%)	0	0	1 (7.1%)
d-TGA	*N* (%)	1(0.3%)	0	1 (12.5%)	0
Valvular aortic stenosis	*N* (%)	8 (2.4%)	2 (0.7%)	2 (0.25%)	4 (28.6%)
Coarctation/shone complex	*N* (%)	4 (1.2%)	0	1 (12.5%)	3 (21.4%)
HLHS	*N* (%)	2 (0.6%)	0	2 (0.25%)	0
Fontan	*N* (%)	3 (0,9%)	3 (1%)	0	0
Tetralogy of Fallot	*N* (%)	1 (0.3%)	0	0	1 (7.1%)
Ebstein´s anomaly	*N* (%)	1 (0.3%)	1 (0.3%)	0	0
s/p mitral valve replacement	*N* (%)	1 (0.3%)	1 (0.3%)	0	0
Pulmonary vein stenosis	*N* (%)	5 (1.5%)	0	2 (0.25%)	3 (21.4%)
Pulmonary atresia	*N* (%)	1 (0.3%)	0	0	1 (7.1%)
Minor complications	*N* (%)	5 (1.5%)	4 (1.3%)	1 (12.5%)	0 (0%)
Major complications	*N* (%)	4 (1.2%)	1 (0.3%)	3 (37.5%)	0 (0%)

*d-TGA* d-Transposition of the great arteries, *HLHS* hypoplastic left heart syndrome, *TSP* transseptal puncture, *VSD* ventricular septal defect

### Transseptal Access to the Left Atrium

Transseptal access to the left atrium was obtained using a Brockenbrough transseptal needle (Brockenbrough BRK, 98 cm, St. Jude Medical/Abbott; Brockenbrough BRK-1, 73 cm St. Jude Medical/Abbott and Brockenbrough BRK-2, 56 cm, St. Jude Medical/Abbott, respectively). Length of the Brockenbrough transseptal needle was selected depending on patients height. For TSP the Brockenbrough transseptal needle was advanced to the tip of a long sheath placed in the superior caval vein (SR-0/SL-1, 63 cm, 8 French (F), St Jude Medical/Abbott or Agilis NXT steerable introducer, Abbot 8.5 F in all subjects with body weight of > 30 kg or ‘Mullins’ Fast-Cath™, 6 to 8 F, St. Jude Medical/Abbott in all subjects with body weight of ≤ 30 kg). All TSP were performed under fluoroscopic guidance using biplane fluoroscopy in right anterior oblique (RAO) 30° and left anterior oblique (LAO) 60° orientation. The sheath-dilator-transseptal needle ensemble (“transseptal system”) was pulled back into the right atrium with the transseptal system tip being directed towards the interatrial septum interrogating the fossa ovalis. The tip was advanced with gentle pressure in a superior-posterior fashion with the tip of the needle being advanced to the tip of the dilator (“blunt” approach). If gentle pressure failed to penetrate the interatrial septum, the tip of the transspetal needle was moved slightly forward in order to penetrate the interatrial septum (“sharp” approach). After the transseptal needle and the tip of the dilator had entered the left atrium, the sheath was pushed over the dilator/needle into the left atrium. The transseptal needle and the dilator were removed subsequently and oxygenated blood was aspirated via the sheath in order to confirm correct position in the left atrium as verified by fluoroscopy. Heparin (100 IE/kg) was administered following TSP in subjects with CHD while it had already been given before TSP in children with normal cardiac anatomy.

Any events that required additional to diagnostic procedures or therapy except standard of care, were considered as major complications while events without need for further treatment were classified as minor complications.

### Statistics

Statistical analysis was performed using SPSS® 26.0 software (IBM, New York, USA). Numerical data is presented as median, interquartile range (IQR) and range. Differences between variables were calculated by Kruskal–Wallis-Test as appropriate. A value of *p* < 0.05 was defined as level of statistical significance.

## Results

All TSP procedures had been guided exclusively by biplane fluoroscopy without additional imaging tools such as transthoracic echocardiography (TTE), transesophageal echocardiography (TEE) or intracardiac echocardiography (ICE). Furthermore, microcatheters and radiofrequency (RF) energy for perforating interatrial septum was not used. TSP could successfully be performed in 323/327 (98.8%) procedures and was abandoned in 4 procedures due to imminent or incurred complications.

For TSP, a 71 cm ‘Brockenbrough’ transseptal needle (BRK-1, St. Jude Medical, St. Paul, MN) was used in 279/327 (85.3%) of procedures while the 56 cm needle (BRK-2, St. Jude Medical, St. Paul, MN) was used in 40/327 (12.2%) procedures and the 98 cm needle (BRK, St. Jude Medical, St. Paul, MN) was used in 8/327 (2.4%) procedures, respectively. Access to the LA was achieved with help of a SR-0/SL-1 sheath in 240/327 (73.4%) procedures and with a Agilis NXT steerable introducer in 8/327 (2.4%) procedures while a ‘Mullins’-sheath was used in 79/327 (24.2%) procedures, respectively.

Main indication for TSP and access to the left atrium was catheter ablation of left sided supraventricular tachycardia (SVT) substrates in 305/327 (93.3%) procedures. In 14/327 (4.3%) procedures with CHD, LA access was required for preoperative diagnostics. In a total of 8/327 (2.4%) procedures in 7 patients, LA access was needed for a catheter intervention except from ablation. All these 7 children were aged < 1 year. Interventions included balloon atrioseptostomy (n = 4), antegrade aortic valvuloplasty (n = 2) and dilatation of pulmonary vein stenosis (n = 2).

**Fig. 1:**
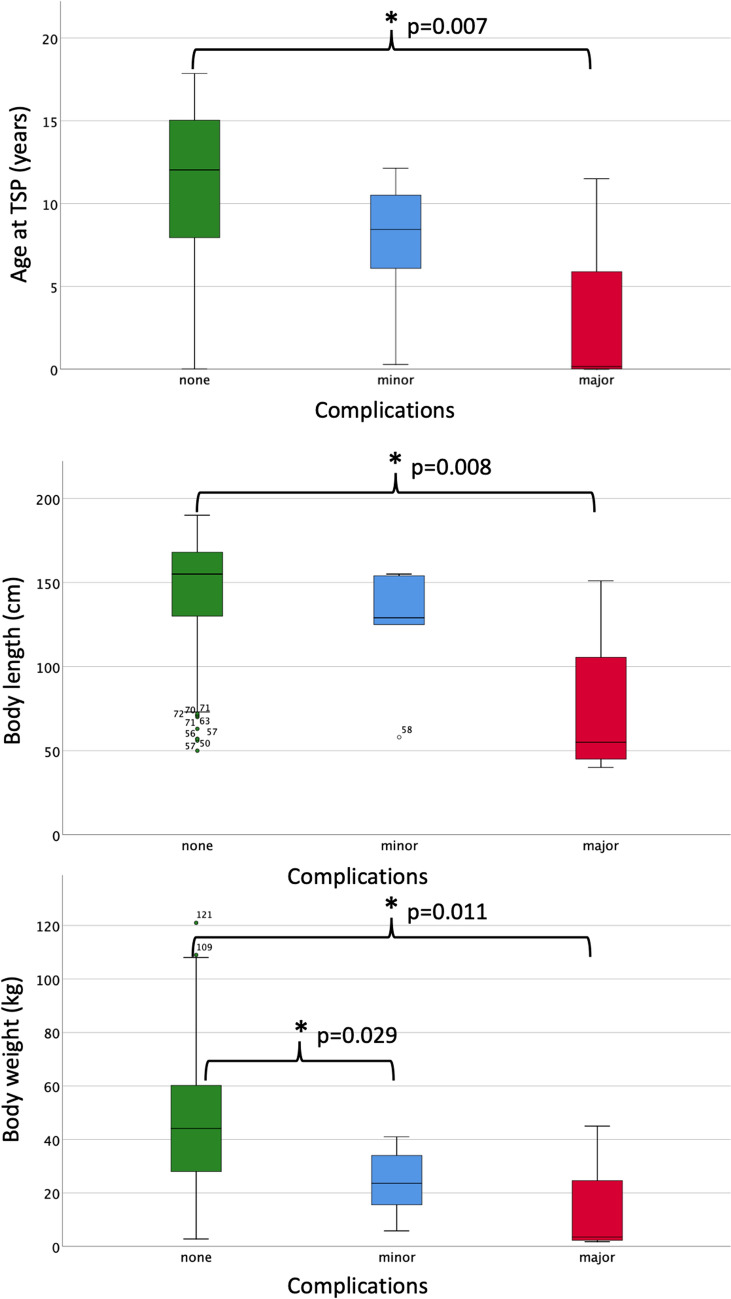
Age, height and body weight at the time of TSP for patients without complications (green box diagram), with minor complications (blue box diagram) and with major complications (red box diagram). Individuals with major complications were significantly younger (*p* = 0.007), smaller (*p* = 0.008) and lighter (*p* = 0.011) than patients without complications at time of TSP. In addition, patients with minor complications had less body weight than patients free from complications (*p* = 0.029)

Major complications occurred in 4/327 (1.2%) of TSP procedures and were substantially more frequent among children with TSP for a hemodynamic intervention (3/8; 37.5%) when compared with TSP for catheter ablation (1/305; 0.3%; *p* < 0.001) or diagnostic procedures (0/14; 0.0%; Table 1). It is of note, that 3/4 major complications occurred in children < 1 year of age (Table 2). Individuals with major complications were significantly younger (*p* = 0.007), smaller (*p* = 0.008) and had less body weight (*p* = 0.011) than patients without complications (Fig. 1). In one newborn with hypoplastic left heart syndrome (HLHS) the restrictive interatrial communication could not be enlarged and the baby developed profound lactate acidosis after TSP without presence of a PE. This baby deceased during the procedure. PE and pericardial tamponade were noted in 2 other babies in whom TSP was needed for ballon atrioseptostomy in d-transposition of the great arteries (d-TGA) and HLHS, respectively. Urgent insertion of an pericardial drainage was needed in both infants. The fourth major complication occurred in an 11-year-old boy with structurally normal heart and SVT due to a left-sided accessory pathway (AP). In this patient TSP failed as the needle crossed the atrial wall causing significant PE with need for pericardial drainage. This patient was discharged in well condition three days after TSP.

**Table 2:** Patients with major complications during TSP

#	Age at TSP (years)	Body weight (kg)	CHD	TSP indication	Complication	PE drainage	Outcome
1	0.0	1.8	HLHS	Creation/enlargement of an interatrial communication	Severe lactate acidosis, circulatory arrest, no PE	None	Deceased during procedure
2	0.0	2.8	HLHS	Creation/enlargement of an interatrial communication	PE after erroneous punction in LA roof, insertion of a drainage, urgent surgical septostomy and bilateral PA-banding same day	Yes	Deceased next day after surgery
3	0.3	4.2	d-TGA	Creation/enlargement of an interatrial communication	PE after erroneous puncture of the pulmonary artery, pericardial tamponade, urgent surgery in the cath lab	Yes	Arterial switch OP two months later
4	11.5	45	None	Ablation of left sided accessory pathway	PE after erroneous puncture of the pulmonary artery, insertion of a drainage	Yes	Discharged well three days after TSP

*CHD* congenital heart defect, *d-TGA* d-transposition of the great arteries, *HLHS* hypoplastic left heart syndrome, *PE* pericardial effusion, *TSP* transseptal puncture

A total of 5/327 (1.5%) minor complications were noted which were uniformly small PEs without need for intervention (Table 3). Patients with minor complications had significantly less body weight than patients without complications (*p* = 0.029, Fig. 1). A total of 4/5 minor PE were noted after ablation of left-sided AP and in one 4-month-old boy after TSP for ballon-valvuloplasy of critical aortic valve stenosis not amenable for retrograde approach. PE resolved spontaneously in all of these cases within 2 days after the procedure and no further action was needed.

**Table 3:** Patients with minor complications during TSP

#	Age at TSP (years)	Body weight (kg)	CHD	TSP indication	Complication	PE drainge	Outcome
5	0.3	5.8	Aortic stenosis	Balloon valvuloplasty of a critical valvular aortic stenosis not amenable for retrograde transaortic approach	Small PE (3–4 mm) after erroneous puncture, no increase of PE, successful second TSP	None	Ross–Konno operation 3 years later
6	6.1	34	None	Ablation of left sided accessory pathway	Small PE (3 mm) after erroneous puncture in pericardial space, no enlargement of the PE and successful second TSP	None	Discharged 3 days later
7	8.4	23.6	None	Ablation of left sided accessory pathway	Erroneous puncture in pericardial space, no PE detectable, no second TSP due to low conduction properties of the left sided AP	None	Discharged 2 days later
8	10.4	15.6	None	Ablation of left sided accessory pathway	Recurrent SVT 4 h after ablation; on Echocardiography small PE (3–4 mm)	None	Redo ablation after 5 months
9	12.1	41	None	Ablation of left sided accessory pathway	Small circular PE (4 mm) after erroneous puncture in pericardial space	None	Discharged 4 days later

*CHD* congenital heart defect, *PE* pericardial effusion, *TSP* transseptal puncture

## Discussion

Transseptal access to the left atrium (LA) is a standard procedure and allows a variety of interventions including atrial fibrillation (AF) ablation and mitral valve repair [1]. The Brockenbrough technique under fluoroscopic guidance and pressure monitoring is safe and effective in adult patients [6]. In infants and children atraumatic passage of the interatrial septum to the LA is often possible via a patent foramen ovale [4, 5, 7]. Safety of fluoroscopic guided TSP in children including those with CHD, however, is yet not well described. Complications were reported to occur in 0.72% procedures in adult patients [7] and in 2.3% in children for RF catheter ablation < 30 kg and normal cardiac anatomy [3]. In contrast to these studies we included subjects with CHD in our analysis. The present study covers results of standardized TSP with use of dual plane fluoroscopy in infants with urgent TSP due to hemodynamic reasons in CHD, TSP for diagnostic procedures in patients with CHD and TSP for ablation of left sided targets. In our patient population including infants and small children, overall, TSP under fluoroscopic guidance was safe and effective with a total of 1.2% major and 1.5% minor complications. There was, however, a significant number of complications (minor complications 12.5%, major complications 37.5%) in babies < 1 year of age and a body weight < 5 kg with urgent TSP due to severe congenital heart defects needing access to the left heart for hemodynamical reasons as well as ballon atrioseptosomy of a restrictive interatrial septum or antegrade valvuloplasty of the aortic valve.

Additional imaging tools were not used in our series. In general, structural abnormities like a thick-/fibrotic or aneurysmatic interatrial septum in patients with CHD may present a challenge for TSP [8]. For these procedures, safety and efficacy of TSP may be improved by additional imaging tools like TTE [8], TEE [9] or ICE [10]. TEE and ICE reflect intracardial anatomy in more detail than TTE or fluoroscopy alone [9, 10]. Both, TEE and ICE, however, are not free from complications and may not be feasible at all due to hemodynamic compromise in critically ill babies needing urgent TSP. In addition, another experienced operator is necessary for TTE or TEE in these situations which may not be available in urgent interventions [11]. TEE associated complications have been reported in approximately 1% of pediatric patients and include transient difficulty in swallowing, shifting the endotracheal tube, transient airway obstruction, transient hoarseness and ventilation problems [12]. ICE is free from risk of esophageal injury and can be performed by the same operator [10]. Yet, ICE needs an additional venous sheath to accommodate a 6–10 F catheter [10] which may increase the risk of vascular complications. Despite these potential complications, TEE and ICE may be helpful additional tools for transseptal puncture in difficult anatomical conditions [10, 12] and maybe an option for successful TSP in small, critically ill babies.

In particular situations the use of a RF guidewire and microcatheter may help in performing a successful TSP. The RF technique utilizes high-frequency electrical current in contrast to mechanical energy using the traditional Brockenbrough approach. RF may be delivered directly via the tip of the transseptal needle for TSP in small CHD patients [8]. In a series of 7 patients with CHD < 1 year of age the procedure was successful in 6/7 infants. Heating the transseptal needle may cause in coring the cardiac tissue into the tip of the open-ended needle with the risk of systemic embolization [13]. Furthermore, size of the hole created by RF was larger than after conventional puncture [14]. Finally, RF puncture inherits a higher risk of bleeding complications by perforating other structures than the interatrial septum e.g. the free atrial wall. Nevertheless, perforating the interatrial septum with a microcatheter, is less invasive than perforating with needle-sheath-system [14].

Microcatheter assisted perforation of the interatrial septum has been shown to be an effective alternative to conventional Brockenbrough approach and feasible with and without RF [14–16]. It is an attractive alternative for perforating the interatrial septum in high risk patients [15, 16].

In contrast to the TSP complications in small babies with CHD, only one severe complication (PE requiring drainage; 0.3%) occurred among the patients receiving TSP for elective ablation of left-sided SVT substrates. This low complication rate of elective TSP is comparable to a previous report with a comparable number of TSP in pediatric electrophysiology procedures [4] and underlines the safety of TSP during elective procedures in children.

In conclusion, access to the left heart was safe and effective in subjects > 1 year of age requiring access to the left heart for elective catheter ablation of left-sided tachycardia substrates or diagnostic procedures in patients with CHD. In contrast to this, TSP was a high-risk procedure in critically ill infants with a restrictive intraatrial septum and need for enlargement of the intraatrial communication. TSP under echocardiographic guidance additional to fluoroscopy as well as perforating the interatrial septum using microcatheter with or without RF may help in performing a successful procedure in individuals with complex interatrial anatomy and thick or tenting interatrial septum. A surgical back-up should be present, whenever an urgent TSP is performed in a small infant with CHD.

## Limitations

This study is limited by its retrospective single center design such as its small number of subjects with CHD and TSP for hemodynamic intervention and TSP for diagnostic purpose. Furthermore, the complication rate of TSP is highly dependent on the operators experience. In our setting emergency TSP in small patients < 1 year of age was exclusively performed by well experienced interventional pediatric cardiologists. Findings may therefore underestimate the real complication rate of TSP in critically ill children with CHD < 1 year of age.

## Abbreviations

AF: Atrial fibrillation
AP: Accessory pathway
CHD: Congenital heart defect
d-TGA: D-Transposition of the great arteries
F: French
HLHS: Hypoplastic left heart syndrome
IQR: Interquartile range
ICE: Intracardiac echocardiography
LA: Left atrium
PE: Pericardial effusion
RF: Radiofrequency
SVT: Supraventricular tachycardia
TEE: Transesophageal echocardiography
TTE: Transthoracic echocardiography
TSP: Transseptal puncture
VSD: Ventricular septal defect
